# Sir2-Independent Life Span Extension by Calorie Restriction in Yeast

**DOI:** 10.1371/journal.pbio.0020296

**Published:** 2004-08-24

**Authors:** Matt Kaeberlein, Kathryn T Kirkland, Stanley Fields, Brian K Kennedy

**Affiliations:** **1**Departments of Genome Sciences and Medicine, University of WashingtonSeattle, Washington, United States of America; **2**Department of Biochemistry, University of WashingtonSeattle, Washington, United States of America; **3**Howard Hughes Medical Institute, University of WashingtonSeattle, WashingtonUnited States of America

## Abstract

Calorie restriction slows aging and increases life span in many organisms. In yeast, a mechanistic explanation has been proposed whereby calorie restriction slows aging by activating Sir2. Here we report the identification of a Sir2-independent pathway responsible for a majority of the longevity benefit associated with calorie restriction. Deletion of *FOB1* and overexpression of *SIR2* have been previously found to increase life span by reducing the levels of toxic rDNA circles in aged mother cells. We find that combining calorie restriction with either of these genetic interventions dramatically enhances longevity, resulting in the longest-lived yeast strain reported thus far. Further, calorie restriction results in a greater life span extension in cells lacking both Sir2 and Fob1 than in cells where Sir2 is present. These findings indicate that Sir2 and calorie restriction act in parallel pathways to promote longevity in yeast and, perhaps, higher eukaryotes.

## Abstract 2

This study indicates that calorie restriction and Sir2 promote longevity in yeast through distinct pathways. This undermines the accepted view, and has implications for aging in higher organisms

## Introduction

The budding yeast Saccharomyces cerevisiae has served as a useful model for aging research, leading to the identification of new longevity genes and pathways whose counterparts can be examined in higher eukaryotes (Kaeberlein et al. 2001). One measure of aging in yeast is the finite replicative life span (RLS) of mother cells, defined as the number of mitotic cycles completed prior to senescence (Mortimer and Johnston 1959). Alternatively, the survival of nondividing yeast cells over time can be monitored and has been termed chronological aging (Fabrizio and Longo 2003). It has been proposed that replicative aging in yeast may be a suitable model for the aging of dividing cells in mammals, such as germ cells; whereas, chronological aging of yeast may be related to the aging of postmitotic tissues.

Replicative aging of yeast can be caused by the accumulation of extrachromosomal rDNA circles (ERCs) in the mother cell nucleus (Sinclair and Guarente 1997), and mutations that decrease ERC formation correlate with increased life span. One example of such a mutation is deletion of the gene encoding the rDNA replication fork barrier protein Fob1, which results in a dramatic decrease in ERC levels accompanied by a 30%–40% increase in mean and maximum RLS (Defossez et al. 1999).

In addition to Fob1, the Sir2 protein has also been found to affect longevity by regulating the rate at which ERCs are formed (Kaeberlein et al. 1999). Sir2 is an NAD-dependent histone deacetylase (Imai et al. 2000; Landry et al. 2000; Tanner et al. 2000) necessary for transcriptional silencing near telomeres (Aparicio et al. 1991), *HM* loci (Ivy et al. 1986; Rine and Herskowitz 1987), and rDNA (Bryk et al. 1997; Smith and Boeke 1997). Deletion of Sir2 increases both rDNA recombination (Gottlieb and Esposito 1989) and ERC formation, while shortening life span by approximately 50% (Kaeberlein et al. 1999). Conversely, overexpression of Sir2 increases life span by 30%–40%. Overexpression of Sir2 in the context of *FOB1* deletion fails to further extend life span, consistent with the idea that Sir2 and Fob1 both impact aging by regulating ERC levels (Kaeberlein et al. 1999).

Calorie restriction (CR) of yeast cells can be accomplished by a reduction in the glucose concentration of growth media from 2% to 0.5% (or lower) and results in a 30%–40% increase in life span (Lin et al. 2000). Several genetic models of CR have also been described. In one model, deletion of the *HXK2* gene, coding for hexokinase, reduces the availability of glucose for glycolysis; while in the others, deletion of other genes, including *gpa2Δ* and *gpr1Δ,* decreases cAMP-dependent protein kinase activity (Lin et al. 2000). Growth in low glucose and the various genetic models of CR have been treated as experimentally interchangeable. While they are clearly not identical, evidence to date suggests that they behave in a similar manner with respect to yeast aging (Lin et al. 2000, 2002, 2004; Kaeberlein et al. 2002, 2004)**.**

Several reports have suggested a link between the enhanced longevity associated with CR and increased activity of Sir2 (Koubova and Guarente 2003). In one genetic model of CR, *cdc25-10* is reported to decrease both rDNA recombination and ERC levels (Lin et al. 2000). In addition, deletion of Sir2 has been shown to prevent life span extension by *cdc25-10* and low glucose (Lin et al. 2000, 2002). These data have been used to support a model whereby CR activates Sir2, thus causing decreased ERC accumulation and increased life span. It was initially proposed that life span extension by CR is the consequence of a metabolic shift resulting in increased cellular NAD available as a substrate for Sir2-dependent histone deacetylation (Lin et al. 2002). More recently, this theory has been supplanted by two competing models for activation of Sir2 by CR: (1) a decrease in cellular nicotinamide (a product inhibitor of Sir2) via upregulation of *PNC1* (Anderson et al. 2003a), and (2) a decrease in cellular NADH (a competitive inhibitor of Sir2) (Lin et al. 2004).

We present here evidence that CR and Sir2 act in different genetic pathways to promote longevity and show that Sir2 is not required for full life span extension in response to CR. In addition, we offer data suggesting that previous experiments were misinterpreted. Finally, we propose a model that reconciles our findings with earlier reports and suggests a greater level of conservation between aging in yeast and higher eukaryotes.

## Results

**Figure 1:**
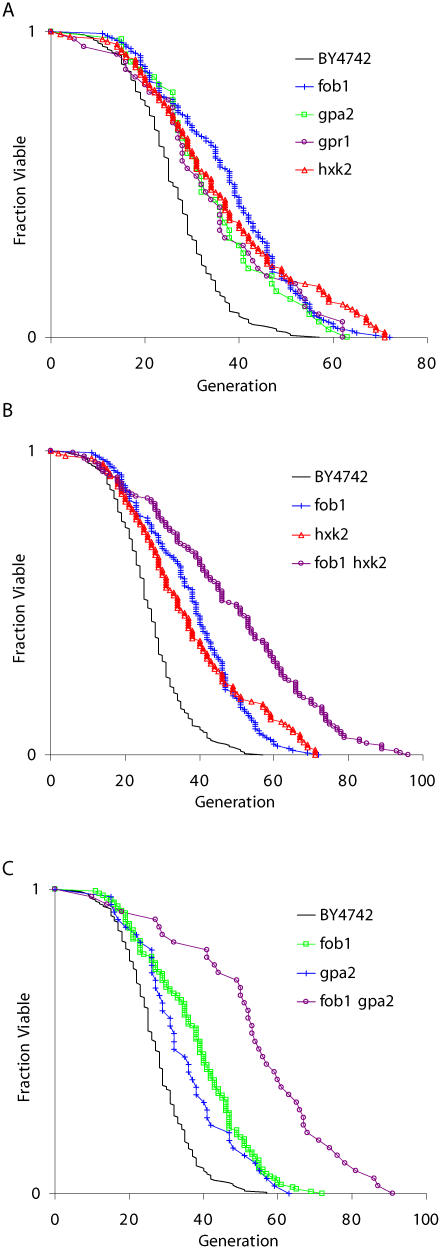
Regulation of Longevity by CR and Fob1 (A) Life span analysis for three genetic models of CR and deletion of *FOB1*. Strains shown (and mean life spans) are BY4742 (26.7), *fob1Δ* (37.8), *gpa2Δ* (34.9), *gpr1* (34.4), and *hxk2Δ* (36.7). (B) *fob1Δ* and *hxk2Δ* increase life span additively. Strains shown (and mean life spans) are BY4742 (26.6), *fob1Δ* (37.3), *hxk2Δ* (36.7), and *fob1Δ hxk2Δ* (48.3). (C) *fob1Δ* and *gpa2Δ* increase life span additively. Strains shown (and mean life spans) are BY4742 (27.2), *fob1Δ* (37.8), *gpa2Δ* (36.7), and *fob1Δ gpa2Δ* (54.5).

We recently carried out a large-scale study of more than 40 single-gene deletions reported to affect aging in yeast (unpublished data). This analysis was performed in the BY4742 genetic background, which has a mean life span significantly longer than most other yeast strains commonly used for aging research (Table 1). Included in this analysis were three genetic models of CR *(hxk2Δ*, *gpa2Δ*, and *gpr1Δ)* and *fob1Δ*. As previously reported for shorter-lived strain backgrounds (Defossez et al. 1999; Lin et al. 2000), each of these single-gene deletions resulted in a 30%–40% increase in life span in BY4742 (Figure 1A).

**Table 1:**
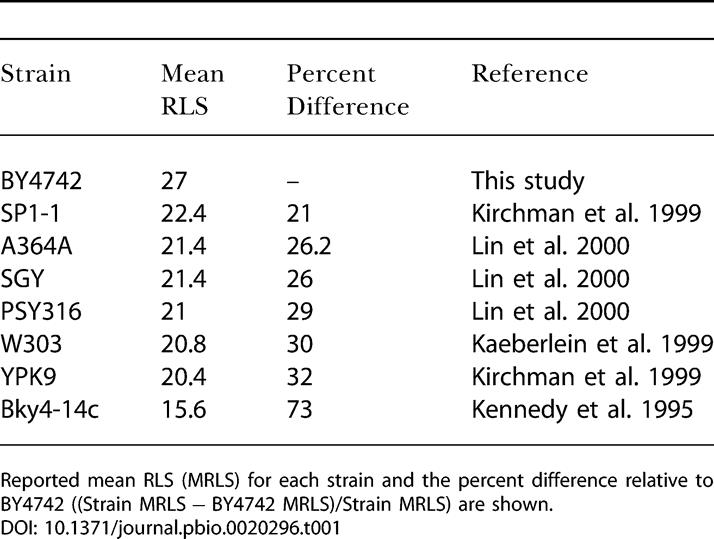
BY4742 Is Long Lived Relative to Other Yeast Strains Commonly Used in Aging Research

Reported mean RLS (MRLS) for each strain and the percent difference relative to BY4742 ((Strain MRLS − BY4742 MRLS)/Strain MRLS) are shown

Since both CR and deletion of *FOB1* increased life span individually in BY4742, we examined the effect of CR combined with deletion of *FOB1*. It is notable that this experiment has not to our knowledge been previously reported. We constructed a *fob1Δ hxk2Δ* double mutant and determined the replicative aging potential of this strain. As expected, both single mutants lived longer than wild-type mother cells (*p* < 0.001). However, the life span of the *fob1Δ hxk2Δ* double mutant greatly exceeded that of either single mutant (*p* < 0.001), suggesting an additional effect on longevity as a result of combining deletion of *FOB1* with CR (Figure 1B).

In order to demonstrate that this synthetic lengthening of life span in combination with *fob1Δ* was not specific to the *hxk2Δ* model of CR, we determined the life span of a *fob1Δ gpa2Δ* double mutant. As observed for *HXK2,* deletion of *GPA2* combined with deletion of *FOB1* resulted in a mean life span significantly greater than was observed for either single mutant (*p* < 0.001), and nearly double that of wild-type cells (Figure 1C). With mean and maximum life spans of 54.5 and 94 generations, respectively**,** the *fob1Δ gpa2Δ* and *fob1Δ hxk2Δ* double mutants are to our knowledge the longest-lived yeast strains reported to date.

**Figure 2:**
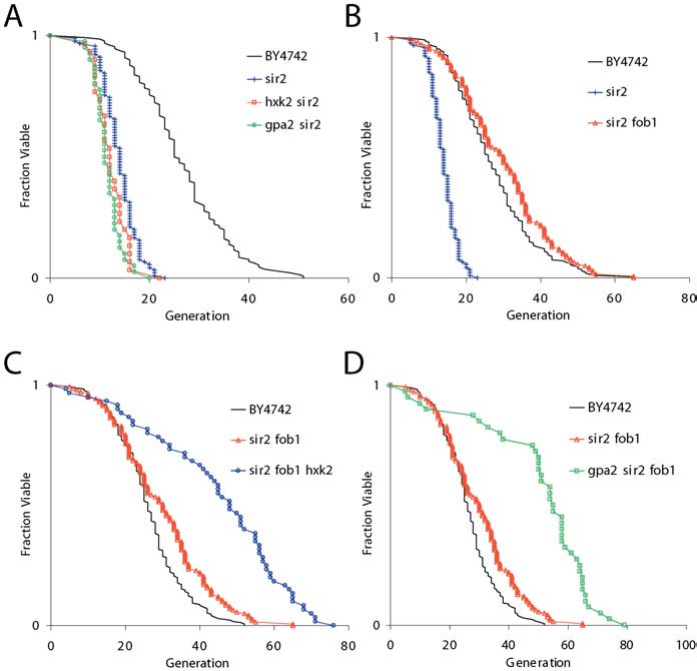
Life Span Extension by CR Does Not Require Sir2 (A) CR fails to increase life span of a *sir2Δ* mutant. Strains shown (and mean life span) are BY4742 (26.7), *sir2Δ* (14.0), *hxk2Δ sir2Δ* (12.4), and *gpa2Δ sir2Δ* (11.7). (B) Deletion of *FOB1* suppresses the short life span of a *sir2Δ* strain. Strains shown and mean life spans are: BY4742 (27.5), *sir2Δ* (14.0), *sir2Δ fob1Δ* (30.0). (C) Deletion of *HXK2* increases the life span of a *sir2Δ fob1Δ* double mutant. Strains shown (and mean life spans) are BY4742 (26.5), *sir2Δ fob1Δ* (30.0), and *sir2Δ fob1Δ hxk2Δ* (45.3). (D) Deletion of *GPA2* increases the life span of a *sir2Δ fob1Δ* double mutant. Strains shown (and mean life spans) are BY4742 (26.6), *sir2Δ fob1Δ* (30.0), and *sir2Δ fob1Δ gpa2Δ* (51.0).

The observation that CR further increases the long life span of a *fob1Δ* mutant is inconsistent with the model that CR increases life span solely by activation of Sir2. Since overexpression of *SIR2* is sufficient to increase the life span of wild-type cells but fails to further extend the life span of a *fob1Δ* mutant (Kaeberlein et al. 1999), CR (acting through Sir2) should also fail to further extend the life span of a *fob1Δ* strain, by this model. Our data therefore suggest the existence of a Sir2-independent pathway by which CR enhances longevity. In order to test this possibility, we determined whether CR would increase life span in the absence of Sir2. As observed in other strain backgrounds, deletion of *SIR2* shortens life span by approximately 50% in BY4742 (Figure 2A), likely because of an elevated level of ERCs (Kaeberlein et al. 1999). Neither deletion of *HXK2* nor deletion of *GPA2* conferred increased life span to the *sir2Δ* mutant. As expected, deletion of *FOB1* was sufficient to suppress the life span defect of cells lacking Sir2 (Figure 2B), consistent with the idea that accelerated ERC accumulation is responsible for the severe life span defect of the *sir2Δ* strain. Surprisingly, in the *sir2Δ fob1Δ* double mutant, deletion of *HXK2* resulted in a robust life span extension (Figure 2C; *p* < 0.001). Similarly, the life span of *sir2Δ fob1Δ gpa2Δ* triple mutant cells was significantly longer than that of *sir2Δ fob1Δ* double mutant cells (Figure 2D; *p* < 0.001). In fact, the life spans of *sir2Δ fob1Δ hxk2Δ* and *sir2Δ fob1Δ gpa2Δ* cells did not differ significantly (*p* ≈ 0.4) from *fob1Δ hxk2Δ* and *fob1Δ gpa2Δ* cells, respectively. Thus, CR clearly enhances longevity in the absence of both Sir2 and Fob1, but not in the absence of Sir2 alone. While seemingly contradictory (see below), these findings demonstrate that Sir2 is dispensable for life span extension by CR, at least in the context of reduced ERC levels (as a result of *fob1Δ*).

**Figure 3:**
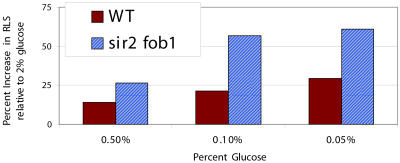
CR Is More Effective at Enhancing Longevity in a *sir2Δ fob1Δ* Double Mutant than in Wild-Type Cells Percent increase in mean life span relative to growth on 2% glucose was determined for 20 mother cells from each strain at 0.5%, 0.1%, and 0.05% glucose.

Genetic models of CR, such as *hxk2Δ* and *gpa2Δ,* have been used as convenient surrogates for CR by growth on low glucose (Lin et al. 2000, 2002); however, it is possible that these genetic models of CR may not completely recapitulate the effects of glucose deprivation. Additionally, unlike genetic models of CR, growth on low glucose provides an opportunity to control the degree of CR by manipulating the glucose concentration within a range of values (Kaeberlein et al. 2002). Taking advantage of this property, we examined the life span of wild-type and *sir2Δ fob1Δ* double mutant cells on 2%, 0.5%, 0.1%, and 0.05% glucose (Figure 3; Figure S1). Wild-type cells showed an increase in mean life span ranging from 15% to 25%, with maximal increases observed at 0.05% glucose (*p* < 0.05). The effect of growth on low glucose was even more pronounced in the *sir2Δ fob1Δ* double mutant, with mean life span increased by 25% on 0.5% glucose (*p* < 0.01) and by 60% on 0.05% glucose (*p* < 0.001).

**Figure 4:**
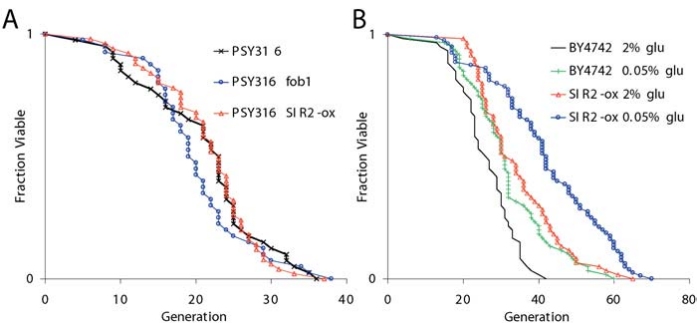
CR Increases the Life Span of Cells Overexpressing *SIR2* (A) Neither deletion of *FOB1* nor overexpression of *SIR2* impact longevity in PSY316. Strains shown (and mean life spans) are PSY316 (21.1), PSY316 *fob1Δ* (20.7), and PSY316 *SIR2*-ox (21.7). (B) Overexpression of *SIR2* and CR increase life span additively in BY4742. Strains shown (and mean life spans) are BY4742 on 2% glucose (26.1), BY4742 on 0.05% glucose (31.8), BY4742 *SIR2*-ox on 2% glucose (34.6), and BY4742 *SIR2*-ox on 0.05% glucose (42.2).

Our data conflict with the report that CR fails to increase the life span of a *sir2Δ fob1Δ* double mutant (Lin et al. 2000). However, all of these prior experiments, as well as nearly all of the published life span data on CR in yeast, were carried out in the PSY316 strain background (Lin et al. 2000, 2002, 2004; Anderson et al. 2002, 2003a, 2003b; Bitterman et al. 2002). We therefore asked whether strain-specific effects might account for this apparent discrepancy. Consistent with prior reports, we observed that growth on low glucose fails to increase the life span of a *sir2Δ fob1Δ* double mutant derived from strain PSY316 (unpublished data). However, the previous experiments demonstrating that either deletion of *FOB1* or overexpression of *SIR2* increase life span were carried out in W303R (Kaeberlein et al. 1999), a genetic background apparently unrelated to PSY316. Notably, life span phenotypes for a *fob1Δ* mutant or *SIR2*-overexpressing strain in PSY316 have not been reported. Thus, we created these strains and measured their life span. Neither deletion of *FOB1* (*p* = 0.29) nor overexpression of *SIR2* (*p* = 0.76) was sufficient to increase life span in the PSY316 background (Figure 4A). In fact, PSY316 behaves differently from the majority of other yeast strains with respect to the roles of *SIR2* and *FOB1* as regulators of longevity, since overexpression of *SIR2* or deletion of *FOB1* has been found to increase longevity in multiple genetic backgrounds, including BY4742 (Table 2).

**Table 2:**
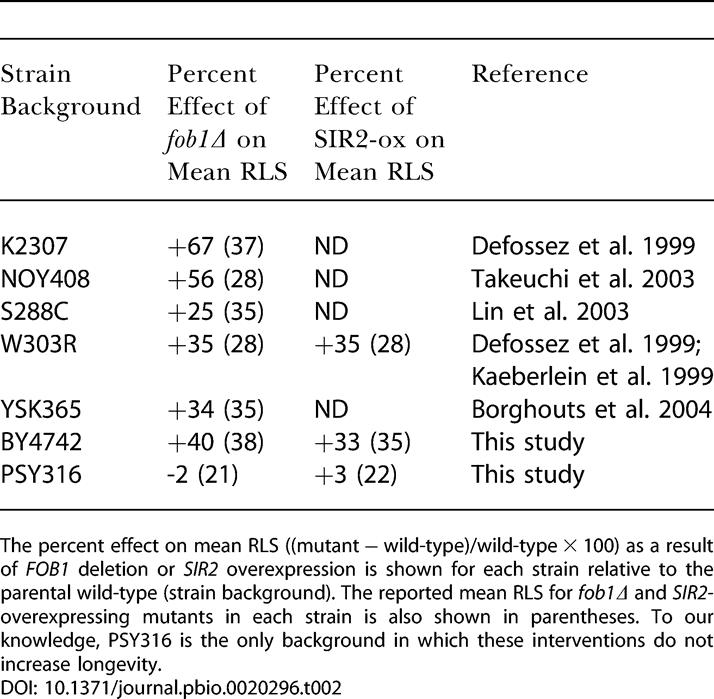
FOB1 Deletion or SIR2 Overexpression Increase Life Span in Multiple Genetic Backgrounds

The percent effect on mean RLS ((mutant − wild-type)/wild-type × 100) as a result of *FOB1* deletion or *SIR2* overexpression is shown for each strain relative to the parental wild-type (strain background). The reported mean RLS for *fob1Δ* and *SIR2-*overexpressing mutants in each strain is also shown in parentheses. To our knowledge, PSY316 is the only background in which these interventions do not increase longevity

**Figure 5:**
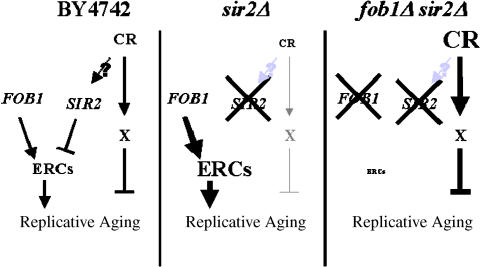
Two Pathways Determine Yeast Longevity The longevity of mother cells can be modified by at least two independent interventions: altered ERC levels and CR. In cells lacking Sir2 but containing Fob1, senescence due to ERCs predominates, causing an extremely short life span that cannot be increased by CR. In cells lacking *FOB1*, ERCs are greatly reduced and the CR pathway predominates. The presence or absence of Sir2 does not impact the longevity benefits of CR under this condition.

Unlike in PSY316, overexpression of *SIR2* in BY4742 significantly increases life span (Figure 4B; *p* < 0.001). Further, growth of *SIR2*-overexpressing cells on low glucose results in an additional life span increase (*p* < 0.001), similar to that observed for *sir2Δ fob1Δ* double mutant cells on low glucose. The observation that CR further enhances the already long life span of cells in which *SIR2* is overexpressed reinforces our model that CR and *SIR2* promote longevity by influencing different pathways (Figure 5).

## Discussion

We present substantial genetic evidence that CR and Sir2 act in different genetic pathways to promote longevity. The combination of CR with *SIR2* overexpression results in an additive life span increase, as expected for two genetic interventions acting in parallel pathways. Further, in the context of *FOB1* deletion, CR results in a larger relative increase in life span in the absence of Sir2 than in cells where Sir2 is expressed. Finally, the ability of CR to promote longevity in a strain lacking Sir2 definitively demonstrates the existence of a Sir2-independent aging pathway responsive to CR.

Experiments have previously suggested that life span extension by CR in yeast is partially Sir2-independent (Jiang et al. 2002). It is important to note, however, that the conditions employed for these experiments involved maintaining the cells on defined medium, which is known to slow growth rate and shorten life span by about 50% (Jiang et al. 2000). Under these conditions, CR is reported to modestly increase mean life span of *sir2Δ* mother cells from seven generations to nine generations. This differs from our results, which demonstrate that CR has no significant effect on life span in the *sir2Δ* background when cells are grown under standard conditions (see Figure 2A). We speculate that the apparently toxic effects of growth on defined medium (as evidenced by dramatically reduced life span and fitness) are partially mitigated by CR in a Sir2-independent manner. It is not clear whether this modest effect (two generations) is related in any way to the robust (20–30 generations) Sir2-independent life span extension caused by CR under standard growth conditions.

The seemingly disparate findings that CR fails to extend the life span of a *sir2Δ*strain (see Figure 2A) but dramatically extends life span in a *sir2Δ fob1Δ* double mutant background (see Figure 2C–2E) can be explained by a model in which there are (at least) two pathways that regulate aging in yeast: one is ERC accumulation and the other is undefined at a molecular level, but responsive to CR (see Figure 5). In our long-lived wild-type background, both processes influence longevity. To explain why CR fails to extend life span in the *sir2Δ* strain, we postulate that the ERC pathway predominates in this mutant. Cells lacking Sir2 exhibit elevated rDNA recombination and increased levels of ERCs (Kaeberlein et al. 1999), resulting in the premature death of nearly all mother cells prior to an age where the CR pathway becomes limiting. Thus, CR, acting through the alternative pathway, fails to impact aging in the *sir2Δ* mutant. In the *fob1Δ* mutant or *sir2Δ fob1Δ* double mutant, strains in which ERCs are greatly reduced (Defossez et al. 1999; Kaeberlein et al. 1999), CR slows aging through the Sir2-independent alternative pathway. This independent pathway should be more important when ERC levels are reduced, and, consistent with this model, we find that CR has a more pronounced effect on life span under these conditions (see Figure 3; Figure S1).

Our findings do not preclude the possibility that CR enhances Sir2 function through previously proposed mechanisms. However, the fact that the life spans of *sir2Δ fob1Δ hxk2Δ* and *sir2Δ fob1Δ gpa2Δ* triple mutants do not differ significantly from those of *fob1Δ hxk2Δ* and *fob1Δ gpa2Δ* double mutants, respectively, suggests that any role for Sir2 in the CR pathway is, at best, minor. Alternatively, it is possible that another protein can substitute for Sir2 as a downstream effecter of CR when Sir2 is absent. This model seems unlikely, however, given a need to postulate that the hypothetical Sir2-like protein could function as a substitute for Sir2 only in a strain lacking Fob1, since CR fails to increase life span in the *sir2Δ* single mutant. The most likely candidate for such a Sir2-like protein is the Sir2 homolog, Hst1. We find that deletion of *HST1* has no effect on life span (unpublished data), suggesting that, at least under normal conditions, Hst1 is not an important determinant of longevity.

Nearly all of the evidence supporting a role for Sir2 in CR-mediated life span extension is derived from experiments carried out in PSY316, further weakening the case for a Sir2-dependent model. The inability of *SIR2* overexpression, in particular, to increase life span in the PSY316 background supports the idea that Sir2 does not play a primary role in CR-mediated life span extension, as it is not straightforward to postulate a model whereby CR would increase life span via activation of Sir2 in a strain background that is insensitive to Sir2 dosage. Further, the inability of the *fob1Δ* mutation to increase life span in PSY316 provides a plausible explanation for why CR is unable to enhance longevity in the PSY316 *sir2Δ fob1Δ* double mutant, and suggests that either deletion of *FOB1* fails to impact ERCs in this background or ERCs are not limiting for life span. While we cannot rule out the possibility that the Sir2-independent nature of CR is unique to BY4742, we note that BY4742 behaves like the majority of other strains with respect to increased life span in response to deletion of *FOB1* or overexpression of *SIR2,* while PSY316 is the only strain (to our knowledge) that is unresponsive to these interventions (Table 2)*.*

The observation that CR further increases the long life span of a *fob1Δ* strain (see Figure 1B and 1C) suggests that the mechanism of enhanced longevity by CR is unrelated to ERCs, as cells lacking Fob1 have dramatically reduced ERC levels. However, it is still possible that ERCs limit the life span of *fob1Δ* cells and that CR slows ERC accumulation by a second pathway that is insensitive to both Fob1 and Sir2. This seems unlikely, since CR is more effective at enhancing longevity in a *sir2Δ fob1Δ* double mutant than in wild-type cells. It has been observed that, while life span is comparable between wild-type and *sir2Δ fob1Δ* cells, ERCs are much reduced in the double mutant (Kaeberlein et al. 1999), suggesting that *sir2Δ fob1Δ* cells are not senescing as a result of ERCs. Thus, life span extension by CR in this context is likely to be unrelated to ERCs.

The existence of an ERC-independent aging pathway in yeast that is modulated by CR is of particular relevance to aging in higher organisms. CR is the only intervention shown to extend life span in a wide range of eukaryotes, including mammals (Weindruch and Walford 1988). In contrast, there is no evidence that ERCs affect aging in organisms other than budding yeast. Nevertheless, in Caenorhabditis elegans, increased expression of the Sir2 ortholog, Sir-2.1, has been found to extend life span in a manner dependent on the Daf-16 transcription factor (Tissenbaum and Guarente 2001). Similarly, the mammalian Sir2 ortholog, SirT1, has recently been reported to regulate the activity of murine Foxo3A (Brunet et al. 2004; Motta et al. 2004)**.** These experiments support a role for Sir2 proteins in eukaryotic aging, linking Sirtuin activity to insulin/IGF-1 signaling. Evidence is accumulating, however, that CR and insulin/IGF-1 act in different pathways to regulate aging in complex eukaryotes. Life span extension by CR is independent of Daf-16 in C. elegans (Lakowski and Hekimi 1998; Houthoofd et al. 2003), and CR can further extend the life span of long-lived insulin/IGF-1 pathway mutants in both C. elegans and mice (Lakowski and Hekimi 1998; Bartke et al. 2001). We present similar evidence that the effects of CR and Sir2 are genetically distinct in yeast, raising the intriguing possibility that aspects of both aging pathways have been conserved.

## Materials and Methods

### 

#### Strains and plasmids.

All yeast strains used in this study are congenic derivatives of BY4742 *(MATα his3Δ1 leu2Δ0 lys2Δ0 ura3Δ0),* except for PSY316AR *(MATα RDN1::ADE2 his3- 200 leu2-3,112 lys2 ura3-52),* PSY316AR *fob1Δ::kanMX,* and PSY316AR *SIR2*-ox. All gene disruptions were verified by PCR. In addition, *sir2Δ* mutants were verified by the sterility phenotype associated with this mutation. Strains overexpressing Sir2 were constructed by genomic integration of an extra copy of *SIR2,* as described (Kaeberlein et al. 1999), and life span was determined for four independent transformants.

#### RLS analysis.

Yeast strains for RLS analysis were removed from frozen stock (25% glycerol, −80 °C) and streaked onto YPD. After 2 d of growth, single colonies were selected and patched to YPD. The next evening, cells were lightly patched to the plates used for life span analysis (4–6 strains per plate). After overnight growth, cells were arrayed onto solid medium using a micromanipulator and allowed to undergo 1–2 divisions. Virgin cells were selected and subjected to life span analysis. Cells were grown at 30 °C during the day and stored at 4 °C at night. Daughter cells were removed by gentle agitation with a dissecting needle and tabulated every 1–2 cell divisions. All life span experiments were carried out on standard YPD plates (2% glucose), except for the low glucose experiments, which were performed on YEP plates supplemented with the indicated amounts of glucose. In order to prevent introduction of bias, strains were coded such that the researcher performing the life span experiment had no knowledge of the strain genotype for any particular strain. For each experiment, each strain was randomly coded at the time of removal from frozen stock. One individual was responsible for assigning codes (K. T. K.) while a different individual (M. K. or B. K. K.) performed the micromanipulation and was unaware of the genotypes of the strains being analyzed.

#### Statistical analysis of data

For statistical analysis, life span datasets were compared using a two-tailed Wilcoxon Rank-Sum test. Mother cell life span and *p*-value matrices for each figure are available in Dataset S1; life span data for individual mother cells are available in Dataset S2. Wilcoxon *p*-values were calculated using the MATLAB ranksum function. Data shown in each figure and used to calculate *p*-values were derived from pair-matched, pooled experiments where each mutant was compared to wild-type cells examined within the same experiment(s). Strains are stated to have a significant difference in life span for *p* < 0.05.

## Supporting Information

Dataset S1 *P-*Value Matrices for Figures 1–4
Each matrix contains the Wilcoxon Rank-Sum *p*-values for a two-tailed test in which the life span data for the strain in the corresponding row were compared against the life span data for the strain in the corresponding column. Significant *p*-values (*p* < 0.05) are colored yellow. *P*-values were calculated using the MATLAB ranksum function.
(46 KB PDF).

Dataset S2 Raw Mother Cell Life Span Data for Figures 1–4
(16 KB TXT).

Figure S1 CR Increases Life Span in Wild-Type and *sir2Δ fob1Δ* Mother Cells (A) Life span extension by CR is maximized at 0.05% glucose in BY4742 mother cells. Mean life spans are shown for cells grown on 2% glucose (24.8), 0.5% glucose (28.3), 0.1% glucose (30.1), and 0.05% glucose (32.1).
(B) Life span extension by CR is maximized at 0.05% glucose in *sir2Δ fob1Δ* mother cells. Mean life spans are shown for cells grown on 2% glucose (26.0), 0.5% glucose (32.9), 0.1% glucose (40.8), and 0.05% glucose (42.0).
(88 KB PS).

### Accession Numbers

The *Saccharomyces* Genome Database (http://www.yeastgenome.org/) accession numbers for the yeast genes and gene products discussed in this paper are *CDC25* (SGDID S0004301), *FOB1* (SGDID S0002517), *GPA2* (SGDID S0000822), *GPR1* (SGDID S0002193), *HST1* (SGDID S0005429), *HXK2* (SGDID S0003222), *PNC1* (SGDID S0003005), and *SIR2* (SGDID S0002200) The LocusLink (http://www.ncbi.nlm.nih.gov/LocusLink/) accession numbers for the non-yeast genes and gene products discussed in this paper are *C. elegans Daf-16* (LocusLink 172981), *C. elegans Sir-2.1* (LocusLink 177924), mouse Foxo3A (LocusLink 2309), and mouse SirT1 (LocusLink 23411).

## Abbreviations

CR: calorie restriction
ERC: extrachromosomal rDNA circle
RLS: replicative life span
