# Supplementary material for: Sir2-Independent Life Span Extension by Calorie Restriction in Yeast
Source: PLoS Biol. 2004 Aug 24;2(9):e296. doi: 10.1371/journal.pbio.0020296 (PMC514491; doi:10.1371/journal.pbio.0020296)
Supplement: Dataset S1 — Each matrix contains the Wilcoxon Rank-Sum p-values for a two-tailed test in which the life span data for the strain in the corresponding row were compared against the life span data for the strain in the corresponding column. Significant p-values (p < 0.05) are colored yellow. P-values were calculated using the MATLAB ranksum function. (46 KB PDF). [file pbio.0020296.sd001.pdf]

This file contains p-value matrices for each figure in Kaeberlein, Kirkland, Fields, and Kennedy. PLOS 2004. Each matrix contains the Wilcoxon Rank-Sum p-values for a 2-tailed test in which the life span data for the strain in the corresponding row is compared against the life span data for the strain in the corresponding column. Significant p-values ($p < .05$) are colored yellow. P-values were calculated using the MATLAB ranksum function.

FIGURE 1B	BY4742	fob1	gpa2	gpr1	hxk2
BY4742		6.33E-14	1.49E-04	1.55E-03	4.11E-08
fob1			1.84E-01	1.45E-01	2.88E-01
gpa2				8.17E-01	6.73E-01
gpr1					4.73E-01
hxk2					

FIGURE 1C	BY4742	fob1	hxk2	fob1 hxk2
BY4742		2.44E-15	5.69E-09	0.00E+00
fob1			2.88E-01	6.54E-06
hxk2				3.68E-06
fob1 hxk2				

FIGURE 1D	BY4742	fob1	gpa2	fob1 gpa2
BY4742		6.57E-14	3.02E-04	1.24E-14
fob1			1.84E-01	2.16E-07
gpa2				3.41E-06
fob1 gpa2				

FIGURE 2A	BY4742	sir2	hxk2 sir2	gpa2 sir2
BY4742		4.37E-26	2.02E-14	1.34E-18
sir2			1.63E-02	1.15E-04
hxk2 sir2				3.87E-01
gpa2 sir2				

FIGURE 2B	BY4742	sir2	sir2 fob1
BY4742		1.95E-28	4.28E-02
sir2			3.96E-25
sir2 fob1			

FIGURE 2C	BY4742	sir2 fob1	sir2 fob1 hxk2
BY4742		1.52E-02	1.43E-12
sir2 fob1			6.02E-09
sir2 fob1 hxk2			

FIGURE 2D	BY4742	sir2 fob1	sir2 fob1 gpa2
BY4742		1.52E-02	3.35E-13
sir2 fob1			9.53E-11
sir2 fob1 gpa2			

FIGURE 3	WT 2%	sir2 fob1 2%	WT 0.5%	sir2 fob1 0.5%	WT 0.1%	sir2 fob1 0.1%	WT 0.05%	sir2 fob1 0.05%
WT 2%		9.89E-01	2.91E-01	3.16E-03	7.58E-02	2.71E-04	1.54E-02	3.85E-04
sir2 fob1 2%			6.07E-01	3.34E-02	1.98E-01	1.16E-03	8.80E-02	1.61E-03
WT 0.5%				3.23E-01	5.60E-01	6.00E-03	4.98E-01	6.50E-03
sir2 fob1 0.5%					3.86E-01	7.84E-02	6.16E-01	4.81E-02
WT 0.1%						1.48E-02	6.26E-01	1.23E-02
sir2 fob1 0.1%							5.46E-02	8.71E-01
WT 0.05%								3.36E-02
sir2 fob1 0.05%								

FIGURE 4A	PSY316	PSY316 fob1	PSY316 SIR2-ox
PSY316		2.96E-01	7.63E-01
PSY316 fob1			1.24E-01
PSY316 SIR2-ox			

FIGURE 4B	BY4742 2%	BY4742 0.05%	SIR2-ox 2%	SIR2-ox 0.05%
BY4742 2%		4.83E-03	2.73E-05	5.64E-11
BY4742 0.05%			2.24E-01	9.35E-06
SIR2-ox 2%				8.06E-04
SIR2-ox 0.05%				
